# Retraction Note: Combined treatment of glibenclamide and CoCl_2_ decreases MMP9 expression and inhibits growth in highly metastatic breast cancer

**DOI:** 10.1186/s13046-025-03311-z

**Published:** 2025-02-04

**Authors:** Zhe Rong, Li Li, Fei Fei, Lailong Luo, Yang Qu

**Affiliations:** 1 Department of Basic Medicine & Experimental Technology, Division of Clinical Medicine, Logistic University of Chinese People’s Armed Police Force, Tianjin, 300162 P. R. China; 2 Department of Pathology, Anhui Medical University, Hefei, 230032 Anhui Province P. R. China; 3 Department of Neurosurgery, Logistic University Affiliated Hospital, Logistic University of Chinese People’s Armed Police Force, Tianjin, 300162 P. R. China

**Retraction Note: J Exp Clin Cancer Res32**,** 32 (2013)**

10.1186/1756-9966-32-32

The Editor-in-Chief has retracted this article. After publication, concerns were raised regarding high similarity among the MMP-9 DMSO, Gibenclamide, and Paclitaxel images in Fig. 2B.

Due to the severity of these concerns in the context of the article, the Editor-in-Chief no longer has confidence in the presented data.

None of the authors have responded to any correspondence from the Publisher about this retraction.

